# The trend of full vaccination coverage in infants and inequalities by wealth quintile and maternal education: analysis from four recent demographic and health surveys in Nepal

**DOI:** 10.1186/s12889-019-7995-3

**Published:** 2019-12-12

**Authors:** Kiran Acharya, Yuba Raj Paudel, Dinesh Dharel

**Affiliations:** 1 New ERA, Rudramati Marga, Kathmandu, Nepal; 2 School of Public Health, University of Alberta, Edmonton, Canada; 3 Cumming School of Medicine, University of Calgary, Calgary, Canada

**Keywords:** Vaccination coverage, Infant, Inequality, Nepal demographic and health survey

## Abstract

**Background:**

Despite policy intention to reach disadvantaged populations, inequalities in health care resource use and health outcomes persist in Nepal. The current study aimed to investigate the trend of full vaccination coverage among infants and its equity gaps between Nepal Demographic and Health Surveys (NDHS) 2001 and 2016**.**

**Methods:**

Using data from NDHS conducted in 2001, 2006, 2011 and 2016, we investigated the trend of coverage of six antigens: Bacille Calmette Guerin (BCG), Diptheria, Pertussis, Tetanus (DPT), Polio, and Measles during their infancy among children aged 12–23 months. We presented trends and correlates of full vaccination coverage by different socio-demographic factors. We measured inequalities in full vaccination coverage by wealth quintile and maternal education using absolute measure (slope index of inequality) and relative measures (Relative index of inequality, concentration index) of inequalities.

**Results:**

Full vaccination coverage among infants steadily increased from 65.6% in 2001 to 87.0% in 2011; however, it decreased to 77.8% in 2016. Province 2 had a significantly lower full vaccination coverage compared to Province1.Although decreasing over time, there were significant inequalities by household wealth quintiles and maternal educational status. The slope index of inequality (SII) for wealth quintiles decreased from − 32.3 [− 45.5,-19.1] in 2001 to an SII of-8.4 [− 18.6,-1.7] in 2016. Similarly, the SII for education decreased from − 61.8 [− 73.5,-50.1] in 2001 to an SII of − 30.5 [− 40.7,-20.2] in 2016. Similarly, the relative index of inequality (RII) also showed an improvement over time, indicating the narrowing equity gap. Additionally, concentration index on full vaccination coverage by wealth quintiles dropped from 0.21 (0.12–0.28) in 2001 to 0.054 (− 0.01–0.12) in 2016. Absolute and relative inequalities were persistently larger by maternal educational status compared to household wealth quintiles throughout the study period.

**Conclusion:**

Full vaccination coverage in Nepal increased from 2001 until 2011 but saw a significant decrement away from the national target after 2011. However, the equity gap by household wealth quintile and maternal education status has narrowed over time. National Immunization programs need to give higher emphasis to infants born to mothers with less education, those born in the poorer wealth quintile households, and those living in Province 2.

## Background

Government of Nepal (GoN) aims to reach all children under-five years of age with vaccines to prevent vaccine-preventable diseases. Immunization services are being provided through a network of static clinics located at health facilities and outreach clinics located in the community [1]. Private and Non-government organization-run health facilities have been increasingly mobilized to provide immunization services from routine immunization clinics as well as during special campaigns [1]. GoN, along with its development partners, has taken various strategies to strengthen immunization services, including full immunization declaration of wards, municipalities, and districts [1]. However, concerns have been raised regarding the sustainability of the full immunization declaration program as being a one-off activity rather than a continuous improvement.

Reaching all children with full immunization services is vital to meet Nepal’s commitment to Sustainable Development Goals. The success of the National Immunization Program (NIP) depends on vaccination coverage, quality of vaccination reporting, and strategies to effectively reach Nepal’s diverse and geographically dispersed population [2]. Nepal Health Sector Strategy 2015–2020 and its implementation plan have a target are to achieve more than 90% full vaccination coverage for children [3]. Literature suggests that national or regional statistics on coverage can mask the underlying inequalities among poor socio-economic groups because overall coverage may be increased when the service coverage increases among well-off groups only, while a simultaneous decline in poor socio-economic groups may often be overlooked [2, 4]. Therefore, it is essential to present disaggregated data on vaccination coverage over time to identify unreached and disadvantaged population groups.

A wealth of evidence suggests that inequalities in child survival outcomes exist mainly by wealth quintiles [5, 6], mother’s educational status [6, 7], and geographical region [8, 9]. However, whether these inequalities are driven by the differences in coverage and service utilization remains poorly understood. A recent study by KC et al. showed an equity gap to have been narrowed in Nepal with increased coverage of immunization services between 2001 and 2014 [10]. However, they used data from different surveys -Demographic and Health Surveys and Multiple Indicators Cluster Survey (MICS), with different methods to compare immunization coverage. Furthermore, the findings might have been subjected to recall bias since they included all eligible children under- five years of age in their analyses, while much of the literature includes only 12–23 months children to measure vaccination coverage [11, 12].

Since household socio-economic position is an important factor determining maternal and child health service utilization in Nepal and elsewhere [12–14],this study aims to report change in equity gaps by household wealth quintile and maternal education using data from most recent four rounds of Nepal Demographic and Health Surveys (NDHS) conducted in 2001, 2006, 2011 and 2016 among children of age 12–23 months. Therefore, the current study has two main objectives. First, we aimed to study the trend of full vaccination coverage by socio-demographic factors. Second, we examined the change in the equity gap in full vaccination coverage by household wealth and maternal education. Findings from this analysis could help policymakers identify disadvantaged groups in terms of immunization service utilization and facilitate policymaking to reduce inequalities and achieve universal health coverage in line with sustainable development goals.

## Methods

### Data source

This study uses data from four NDHS conducted in 2001, 2006, 2011, and 2016, which obtained comparable nationally representative samples of children age 12–23 months. DHS is an extensive survey that covers a variety of indicators, including vaccination coverage among children age 12–23 months, and has a rigorous design, [15]. The DHS sample is selected in two stages. The first stage involves selecting clusters with probability proportional to size from a national master sample frame. In the second stage, a systematic sample of households is drawn from a listing of households in each of the DHS clusters. NDHS 2001, 2006, and 2011 were selected in two stages, while the 2016 NDHS sample was stratified and selected in two stages in rural areas and three stages in urban areas [16–19].

### Measures

Full vaccination refers to having a vaccination against tuberculosis; i .e.Bacille Calmette Guerin (BCG), three doses of combination vaccine including at least diphtheria, pertussis, and tetanus (DPT), three doses of oral polio vaccines, and a dose of measles-containing vaccine by the age of 12 months. The full vaccination coverage is calculated based on vaccination data collected from mothers of children age 12–23 months. If vaccination cards were not available at selected households at the time of the survey, interviewers relied on mother’s reports to determine vaccination records. Predictor variables used to assess disparities included: mothers’ education (i. no education, ii. primary, iii. Secondary, and iv. higher education), wealth quintiles (i. poorest, ii. Poorer, iii. Middle, iv. Richer, and v. richest), sex of the child (i. male and ii. female), caste/ethnicity (i. Brahmin/Chettri, ii. Terai/Madhesi other, iii. Dalits, iv. Newar/Janajatis and v. Muslims/others), place of residence (i. rural and ii. urban), province (1to7) and ecological zone (i. Mountain, ii. Hills, and iii. Terai). These variables were taken from the available literature [10, 20, 21] and were used to demonstrate the disaggregated rates of full immunization coverage in Nepal. We showed trend by provinces instead of previously adopted political division based on development region because the new constitution of Nepal endorsed on September 2015, devolved the power into three levels of government: one federal level, seven provinces, and 753 local governments. Recently conducted NDHS 2016, stratified each province into urban and rural areas, yielding 14 sampling strata [18]. For previous surveys, we first identified the cluster numbers and recorded them with the current administrative division of each province. We verified the comparability of clusters across each survey using Global Positioning System (GPS) code.

Given the difficulty of collecting income and expenditure data in developing countries, DHS surveys collect data on household ownership of consumer goods, dwelling materials, sources of drinking water, types of sanitation facilities, and other characteristics that relate to economic status. With this data, an index score is computed for each household using principal component analysis. The entire sample is then ranked according to this score and is divided into quintiles, from the first quintile (Q1)—the poorest 20% of the household population—to the fifth quintile (Q5)—the wealthiest 20% [22]. Wealth quintile ranking indicates relative rather than the absolute economic status of the household. The bottom 20% measured in the 2001 NDHS may not have the same absolute level of wealth as the bottom 20% measured in the 2006, 2011 and 2016 NDHS. In other words, wealth status is not comparable across surveys and countries. This study, however, is not affected by this limitation because it focused on the disparities in vaccination coverage between the wealthy and the poor only within each survey.

### Statistical analysis

Analyses were performed using the full national sample of children age 12–23 months. We accounted for the DHS complex sampling design when estimating SEs through the use of Stata’s ‘svy’ commands (Stata 15.0) for survey data.

### The trend of full vaccination coverage

Time trends were examined by presenting vaccination coverage and 95% CI by background characteristics to assess how coverage was changing in different strata during the study period. We also conducted bivariate and multivariate logistic regression analysis on a pooled sample of children aged 12–23 months (*n* = 4330) from four DHS surveys to identify significant correlates of full vaccination coverage. Logistic regression analysis also allowed us to investigate whether full immunization coverage was significantly changing over survey years compared to 2001.

### Measurements of the equity gap

Our objective to investigate inequalities in full vaccination coverage by maternal education and wealth quintile based on evidence from literature was corroborated by our multivariate logistic regression analysis on the current pooled sample from NDHS 2001–2016, which showed that these two variables were the strongest correlates of full vaccination coverage (Table 3). We calculated measures for both absolute and relative inequalities over time. To assess inequalities, we ranked children age 12–23 months using the wealth index and allotted a relative ranking based on their position in the cumulative distribution. We used logistic regression models to estimate the association between the children’s relative rank and vaccination coverage. The SII and RII were obtained using marginal effects and nlcom post-estimation commands. We expressed inequalities in terms of the slope index of inequality (SII) and the relative index of inequality (RII). The SII expresses the absolute percentage point difference in vaccination coverage between the predicted lowest social rank and highest rank (in wealth and education distribution), assuming a linear relationship between social rank and vaccination coverage [24]. The RII expresses the ratio of the predicted outcomes between the two extremes of the social rank, assuming a log-linear relation [24]. RII = 1 indicates equality, whereas any value of RII below or above 1 indicates inequality.

We also calculated the concentration index (CI), which quantifies the degree of economic inequality using information from five wealth quintiles. Therefore, it is a composite summary of inequality across the entire population. The value of CI ranges from − 1 to + 1 with a value of 0 indicating perfect equality and positive values indicating health service utilization concentrated among rich and negative values indicating service utilization concentrated among poor [25].

### Ethics

DHS surveys were conducted after receiving ethical approval from the Nepal Health Research Council (NHRC) ethical review committee. All of the respondents were informed of the purpose of the survey and were informed that participation was not compulsory and that if they did choose to participate, they were assured of the confidentiality of the information. Written consent was sought before beginning the interview as per NHRC ethical review guidelines. Separate ethical approval was not needed for this analysis.

## Results

There were a total of 1313 children aged 12–23 months in 2001 NDHS sample, 984 in NDHS 2006, 1000 in NDHS 2011 and 1034 in NDHS 2016 giving a total of (*N* = 4330) children in the pooled sample. Trend data showed that there had been considerable socio-demographic changes in Nepal over the last fifteen years (Table 1). The proportion of respondents dwelling in urban areas increased from 6% in 2001 to 54% in 2016. The percentage of mothers with no formal education decreased from 71% in 2001 to 31% in 2016. There has been little change in proportion of respondents from Mountain areas, proportion of respondents from Hills have decreased from 43.0% in 2001 to 37.7% in 2016. Whereas, proportion of respondents from Terai region have increased from 49.8% in 2001 to 55.0% in 2016. Male were oversampled in 2006 and 2016 NDHS compared to female children. Female children were slightly oversampled in 2001 whereas there was almost equal proportion in 2011.

**Table 1:** Trend in socio-demographic characteristics of children aged 12–23 months born in the last five years of NDHS 2001,2006, 2011 and 2016

	2001 (95% CI) N = 1313	2006 (95% CI) *N* = 984	2011 (95% CI) *N* = 1000	2016 (95% CI) *N* = 1034	*P*-value (Chi-square test)
Place of residence					
Urban	6.6 (5.0–8.6)	12.3 (10.3–14.7)	9.7 (8.2–11.5)	54.5 (48.4–60.5)	< 0.0001
Rural	93.4 (91.4–95.0)	87.7 (85.3–89.7)	90.3 (88.5–91.8)	45.5 (39.5–51.6)	
Ecological region					
Mountain	7.2 (5.9–8.7)	9.9 (8.0–12.3)	7.5 (6.3–8.9)	7.2 (4.9,10.7)	0.1762
Hill	43.0 (39.5–46.4)	43.0 (38.9–47.3)	40.2 (35.4–45.2)	37.7 (32.2–43.5)	
Terai	49.8 (46.2–53.4)	47.0 (42.9–51.3)	52.3 (47.0,57.6)	55.0 (49.4–60.6)	
Province					
Province 1	19.1(15.9–22.8)	17.4 (14.1–21.2)	20.0 (15.3–25.7)	16.4 (13.9–19.2)	0.1181
Province 2	21.2(17.0–26.0)	17.2 (13.1–22.3)	23.9(16.9–32.5)	25.1 (21.9–28.5)	
Province 3	15.1(12.1–18.6)	16.7 (12.9–18.1)	13.5 (10.3–17.6)	16.3 (12.8–20.5)	
Province 4	8.2(5.6–11.7)	12.4(8.4–18.1)	10.6 (6.8–16.2)	9.1 (7.6–10.7)	
Province 5	16.1(12.0–21.3)	14.4(11.3–18.1)	15.0(11.0–20.1)	19.0 (16.0–22.3)	
Province 6	9.7(6.6,14.0)	6.6 (4.2,10.3)	6.9 (4.7,10.0)	6.1 (5.1,7.3)	
Province 7	10.7(8.8,13.0)	15.3 (11.8,19.5)	10.1 (8.3,12.2)	8.2 (6.7,9.9)	
Sex of child					
Male	48.1(45.2,51.0)	52.3 (48.8,55.8)	50.1 (46.4,53.8)	55.8 (52.5,59.1)	0.0080
Female	51.9(49.0,54.8)	47.7 (44.2,51.2)	49.9 (46.2,53.6)	44.2 (40.9,47.5)	
Educational level of mother					
No education	71.0 (67.2–74.4)	56.3(51.7–60.7)	45.2 (39.4–51.2)	31.1 (27.2–35.2)	< 0.0001
Primary	14.6 (12.6–16.8)	18.9(15.9–22.4)	20.0 (16.6–23.8)	20.5 (17.6–23.8)	
Secondary	10.1 (8.2–12.3)	18.2(15.2–21.6)	21.1 (17.4–25.3)	25.8 (22.4–29.5)	
Higher	4.4 (3.2–6.0)	6.6(4.7–9.2)	13.7 (11.0–16.9)	22.6 (19.0–26.7)	
Wealth quintile					
Poorest	26.1 (22.7–29.9)	25.8(21.6–30.6)	24.8 (20.8–29.1)	20.8 (17.2–24.8)	0.4163
Poorer	22.5 (19.7–25.6)	20.2(16.7–24.3)	22.7(19.0–26.8)	22.1 (19.0–25.6)	
Middle	19.3 (16.4–22.6)	20.8(17.2–25.0)	21.7(17.6–26.6)	22.9 (19.8–26.4)	
Richer	17.3 (14.8–20.2)	17.2(13.7–21.3)	18.3 (15.2–21.8)	21.8 (18.5–25.6)	
Richest	14.7 (11.8–18.3)	15.9(12.6–19.9)	12.6 (9.4–16.6)	12.4 (9.7–15.7)	
Caste/Ethnicity					
Brahamin/Chhetri	29.2 (25.1–33.8)	32.3(27.7–37.3)	26.7 (22.2–31.7)	25.8 (22.1–29.8)	0.1749
Terai/Madhesi other	15.6 (12.1–19.9)	8.6(6.0–12.1)	11.1 (7.2–16.8)	20.2 (16.2–24.9)	
Dalits	14.3 (11.2–18.0)	15.0(11.7–18.9)	16.9 (12.8–21.9)	15.5 (12.5–19.0)	
Janajati/Newar	34.8 (29.6–40.3)	37.5(31.7–43.7)	36.2 (29.2–43.7)	30.9 (26.5–35.6)	
Muslim/Other^1^	6.1 (3.2–11.2)	6.6(3.3–12.7)	9.1 (3.8–20.4)	7.6 (5.0–11.5)	

^1^Other includes Marwari, Bangali, Jain, Punjabi/Sikh, and Unidentified others

### The trend of full vaccination coverage

Table 2 shows that the full vaccination coverage steadily increased from 65.6% in 2001 to 87.0% in 2011, but it decreased to 77.8% in 2016. Table 3 showed that there was a significant increase in coverage over the NDHS years compared to 2001. The urban/rural differential in full vaccination decreased from almost 9% in 2001 to less than 2% in 2016(Table 2), and the difference did not reach statistical significance with adjusted Odds Ratio (OR) 1.4 (95% CI 0.99–1.8) in a multivariate analysis (Table 3). Terai region in Nepal had a lower coverage of full vaccination compared to Hills and Mountains, but the difference did not reach statistical significance. Similarly, Province 2 had consistently low coverage with less than two-thirds of infants being fully immunized in 2016, and province 2 had a significantly lower coverage of full vaccination compared to province 1 in the multivariate model. The rate of full vaccination coverage showed a clear increment with increasing maternal education in all NDHS years, being 57% in infants of mothers with no education to 90.9% among those with higher education in 2001 and from67.8 to 91.2% in 2016(Table 2), respectively. Infants born to mothers with primary or higher education had higher chances of being fully immunized compared to infants born to mothers with no education after adjusting for other socio-demographic variables. Similarly, infants born in households with higher wealth quintiles had higher chances of being fully immunized compared to infants born in the poorest wealth quintile households in a multivariate model. Interestingly, the drop in full vaccination coverage in 2016 from the level of 2011 was higher among the middle and the richest income quintile compared to the poorest income quintile.

**Table 2:** Trend of full vaccination coverage by socio-demographic characteristics of children aged 12–23 months (2001–2016)

	2001 (95% CI) *N* = 1313	2006 (95% CI) *N* = 984	2011 (95% CI) N = 1000	2016 (95% CI) N = 1034
Place of residence				
Urban	74.9 (63.5,83.6)	86.3 (77.1–92.2)	90.0 (84.2–93.8)	78.5 (73.3–83.0)
Rural	65.0 (59.5,70.1)	82.4 (77.8–86.1)	86.6 (81.4–90.5)	77.0 (71.7–81.6)
Ecological region				
Mountain	63.5 (50.6,74.6)	71.3 (55.5–83.2)	88.2 (76.8–94.4)	74.1 (64.1–82.1)
Hill	70.4 (62.1,77.6)	81.6 (74.3–87.2)	89.5 (84.6–92.9)	88.0 (83.6–91.4)
Terai	61.8 (54.5,68.5)	86.4 (81.4–90.3)	84.8 (76.6–90.5)	71.3 (66.1–76.0)
Province				
Province 1	76.3(67.8,83.1)	84.7 (77.2–90.1)	87.3 (75.7–93.0)	79.4 (72.4–85.1)
Province 2	51.3(40.8,61.8)	79.2 (68.5–87.0)	79.3 (64.8–88.8)	65.2 (56.4–73.0)
Province 3	72.6(60.2,82.3)	80.6 (63.9–90.7)	91.3 (83.0–95.8)	85.3 (74.7–91.9)
Province 4	69.9(53.7,82.3)	89.5 (80.1–94.8)	92.6 (75.2–98.1)	92.7 (86.5–96.2)
Province 5	64.3(49.8,76.6)	87.0 (79.2–92.1)	91.0 (86.0–94.3)	78.3 (68.8–85.5)
Province 6	70.0(42.7,87.9)	76.8 (60.4–87.8)	76.5 (60.1–87.5)	74.9 (64.7–82.9)
Province 7	59.7(48.2,70.2)	80.5 (67.6–89.1)	93.7 (88.1–96.8)	83.4 (74.9–89.4)
Sex of child				
Male	67.5(62.1,72.4)	84.9 (80.3–88.6)	88.2 (82.8–92.1)	77.4 (72.8–81.5)
Female	63.9(57.6,69.7)	80.6 (75.6–84.8)	85.7 (80.5–89.7)	78.4 (74.2–81.1)
Educational level of mother				
No education	57.0(51.0,62.9)	74.3 (68.2–79.6)	78.1 (70.0–84.4)	67.8 (61.0–73.9)
Primary	83.2(75.6,88.7)	88.2 (81.4–92.7)	94.6 (88.6–97.5)	75.8 (68.2–82.1)
Secondary	89.6(82.8,93.9)	97.7 (94.6–99.1)	95.2 (89.4–97.9)	79.8 (74.0–84.6)
Higher	90.9(79.0,96.4)	99.0 (95.5–99.8)	92.4 (85.1–96.3)	91.2 (86.7–94.3)
Wealth quintile				
Poorest	54.2 (45.5,62.6)	68.0 (58.5–76.2)	84.5 (78.0–89.4)	76.6 (69.7–82.4)
Poorer	62.4 (53.5,70.5)	82.4 (73.9–88.5)	83.9 (75.8–89.6)	77.2 (69.6–83.3)
Middle	64.5 (55.3,72.8)	87.1 (79.9–92.0)	84.0 (72.4–91.4)	70.9 (63.7–77.2)
Richer	74.7 (66.8,81.3)	90.7 (84.0–94.7)	91.5 (83.4–95.9)	84.8 (78.2–89.7)
Richest	81.6 (73.5,87.6)	93.5 (85.7–97.2)	95.7 (88.8–98.4)	81.6 (70.8–89.1)
Caste/Ethnicity				
Brahamin/Chhetri	74.9 (64.9,82.8)	87.7 (81.2,92.2)	90.7 (85.3,94.3)	87.3 (82.4,91.0)
Terai/Madhesi other	51.1 (39.1,63.0)	77.9 (63.9,87.5)	82.0 (67.9,90.7)	64.3 (55.3,72.5)
Dalits	58.9 (48.3,68.7)	70.6 (59.1,80.0)	85.7 (76.4,91.7)	73.2 (65.1,80.0)
Janajatis/Newar	71.4 (64.3,77.6)	85.2 (77.7,90.5)	93.5 (88.4,96.4)	83.3 (77.8,87.6)
Muslim/Other	41.0 (19.1,67.1)	79.6 (64.4,89.4)	58.5 (48.9,67.5)	69.0 (56.9,78.9)
Total	65.6 (60.5–70.4)	82.8 (78.8–86.3)	87.0 (82.3–90.6)	77.8 (74.2–81.1)

**Table 3:** Socio-demographic correlates of vaccination coverage among children age 12–23 months (2001–2016 Pooled data)

	Full vaccination coverage (N = 4330)	
	Unadjusted OR(95% CI)	Adjusted OR(95% CI)
Survey Year		
2001	ref.	ref.
2006	2.5*** (1.7–3.6)	2.3*** (1.7–3.3)
2011	3.5** (2.3–5.3)	3.1*** (2.0–4.6)
2016	1.8* (1.4–2.5)	1.4 (0.9–1.9)
Place of residence		
Urban	ref.	ref.
Rural	0.8 (0.6–1.0)	1.4 (0.99–1.8)
Ecological region		
Mountain	ref.	ref.
Hill	1.6* (1.1–2.3)	1.3 (0.9–2.0)
Terai	1.1 (0.7–1.5)	1.2 (0.8–1.8)
Province		
Province 1	ref.	ref.
Province 2	0.5*** (0.3–0.7)	0.6** (0.4–0.8)
Province 3	1.0 (0.6–1.5)	1.0 (0.7–1.6)
Province 4	1.4 (0.8–2.3)	1.1 (0.7–1.8)
Province 5	0.8 (0.6–1.2)	0.8 (0.5–1.2)
Province 6	0.6 (0.4–1.1)	1.1 (0.6–2.0)
Province 7	0.8 (0.5–1.2)	1.1 (0.7–1.6)
Educational level of mother		
No education	ref.	ref.
Primary	2.8*** (2.2–3.7)	1.7 (0.9–3.1)
Secondary	4.3*** (3.2–5.7)	3.4*** (1.7–6.5)
Higher	6.1*** (4.1–8.9)	2.4* (1.1–5.9)
Wealth quintile		
Poorest	ref.	ref.
Poorer	1.4* (1.1–1.7)	1.6** (1.2–2.0)
Middle	1.4* (1.1–1.8)	1.6** (1.2–2.2)
Richer	2.5*** (1.81–3.4)	2.4*** (1.7–3.3)
Richest	3.2*** (2.2–4.6)	2.2*** (1.4–3.3)

Variables that showed *p* > 0.1 in bivariate analysis were dropped in multivariate analysis (23),except wealth quintile and place of residence were retained being important variables for our study

***P < 0.001, **p < 0.01, *p < 0.05; ref. means reference category

### Equity gap in full vaccination coverage

**Fig. 1:**
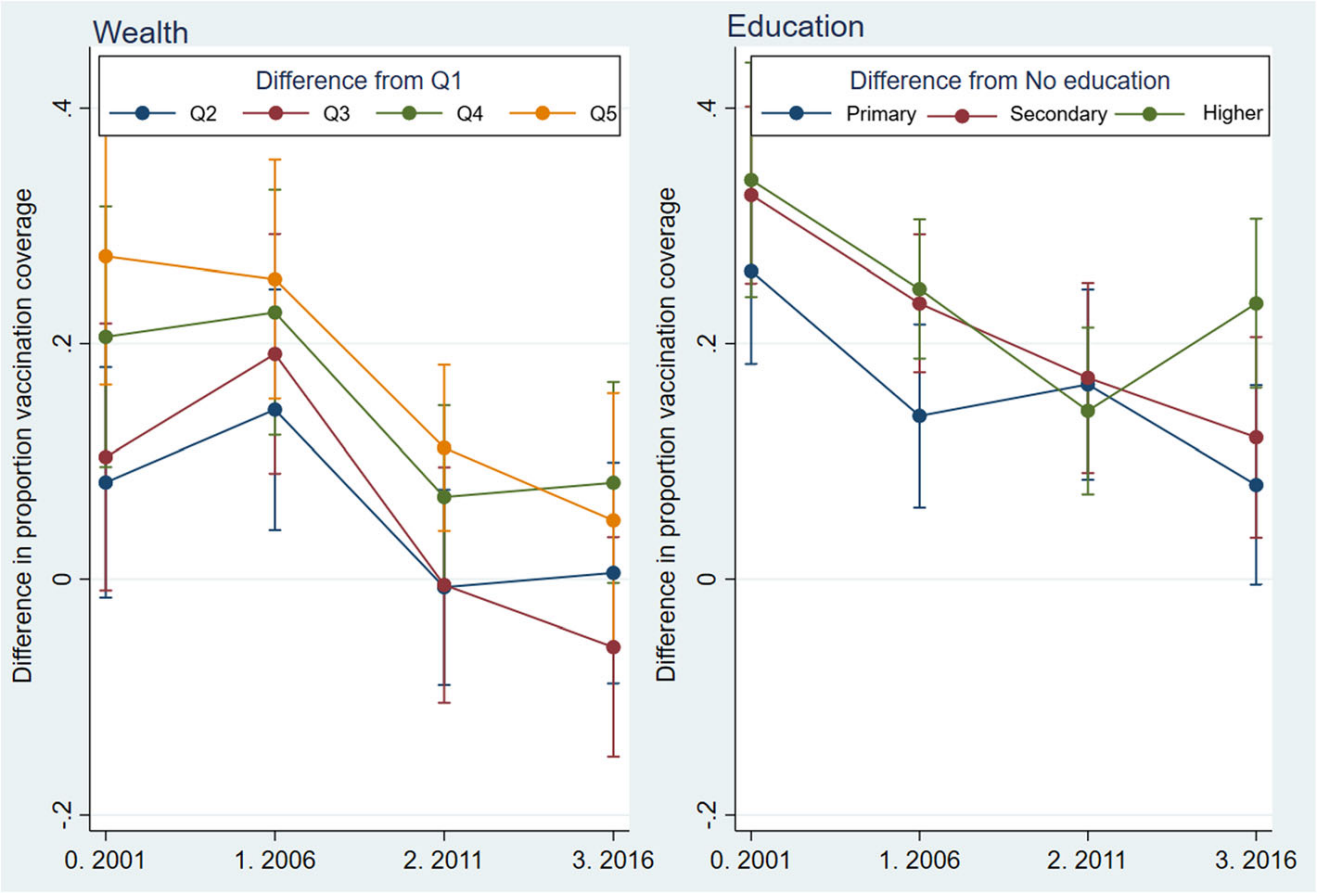
Crude prevalence difference in full vaccination coverage (2001–2016) by wealth and education

The absolute difference in predicted full vaccination coverage between the richest income quintile and the poorest quintile significantly decreased between 2001 and 2016, from an SII of-32.3 [− 45.5,-19.1] to an SII of-8.4 [− 18.6,-1.7] (Table 4). Likewise, the absolute difference in predicted coverage between infants born to mothers with no education and those born to mothers with higher education also dropped from and SII of − 61.8 [− 73.5,-50.1] in 2001 to an SII of − 30.5 [− 40.7,-20.2] in 2016. However, the crude difference between those with no education and those with higher education increased to 23.4% in 2016 compared to 14.3% in 2011 (Fig.1). Similarly, the crude difference in full vaccination coverage with respect to the lowest wealth quintile decreased over time for all other wealth categories except the middle wealth category in 2016. Infants born to middle-income quintile- in fact- had nearly 6% lower full vaccination coverage compared to infants born to the poorest income quintile in 2016. SII was higher for educational status compared to wealth quintiles for all surveys (Fig.1). Furthermore, SII for wealth quintiles declined to a greater extent than for maternal educational status.

**Table 4:** Crude differences and Slope Index of Inequality (SII) for vaccination coverage among 12–23 months’ children by maternal education and household wealth quintile (2001–2016)

	2001	2006	2011	2016	P-value*
Rate difference (95% CI)					
No education (ref.)					
Primary	26.2 (18.3,34.0)	13.9(6.1,21.6)	16.5 (8.5,24.6)	8.0(−0.5,16.5)	0.0184
Secondary	32.6 (25.1,40.1)	23.4 (17.6,29.3)	17.1 (9.0,25.2)	12.0(3.5,20.6)	0.0023
Higher education	33.9 (24.0,43.8)	24.6 (18.7,30.5)	14.3 (7.2,21.4)	23.4 (16.3,30.6)	0.0136
SII (95% CI)	−61.8 (−73.5,-50.1)	−47.9(−61.2,-34.6)	−29.8 (−43.7,-15.8)	−30.5 (−40.7,-20.2)	< 0.0001
Rate difference (95% CI)					
Poorest (ref.)					
Poorer	8.2(−1.6,18.0)	14.4 (4.2,24.6)	−0.7 (−9.0,7.6)	0.5 (−8.8,9.9)	0.0970
Middle	10.4 (−1.0,21.7)	19.1 (9.0,29.3)	−0.5(− 10.5,9.5)	−5.8(− 15.1,3.6)	0.0023
Richer	20.6(9.5,31.6)	22.7(12.3,33.1)	7.0 (−0.8,14.8)	8.2 (−0.3,16.7)	0.0355
Richest	27.4 (16.5,38.3)	25.5 (15.3,35.6)	11.2 (4.1,18.2)	5.0(−5.8,15.8)	0.0040
SII (95% CI)	−32.3 (−45.5,-19.1)	−32.8 (−45.7,-19.9)	−12.6 (−21.6,-3.6)	−8.4 (− 18.6,-1.7)	< 0.0001

*P value for test of heterogeneity in rate difference or SII across years

Similarly, the relative Index of Inequality also showed an improvement over time. The ratio of predicted coverage between those born to mothers with no education to those born to mothers with higher education increased from 0.3 [0.2,0.5] in 2001 to 0.7 [0.6,0.8] in 2016 (Table 5). Similarly, the ratio of predicted coverage between the lowest wealth quintile to the highest wealth quintile or RII also showed an improvement from 0.6 [0.5, 0.8] in 2001 to 0.9 [0.8,1.0] in 2016. Furthermore, the Concentration Index, which is a composite measure of inequality encompassing all wealth quintiles, dropped from 0.21 (0.12–0.28) in 2001 to 0.054 (− 0.01–0.12) in 2016 reflecting a reduction in pro-rich inequality (Table 5). The relative inequalities were larger by educational status compared to wealth quintiles for all DHS surveys.

**Table 5:** Relative Index of Inequality (RII: wealth and education), and Concentration Index (wealth) for vaccination coverage among 12–23 months’ children by maternal education and household wealth quintile, Nepal Demographic and Health Surveys (2001–2016)

	2001	2006	2011	2016	*P* value*
Coverage ratio(95% CI)					
No education (ref.)					
Primary	1.5 (1.3,1.6)	1.2 (1.1,1.3)	1.2 (1.1,1.3)	1.1 (1.0,1.3)	0.0103
Secondary	1.6 (1.4,1.8)	1.3 (1.2,1.4)	1.2 (1.1,1.3)	1.2 (1.0,1.3)	0.0018
Higher education	1.6 (1.4,1.8)	1.3 (1.2,1.4)	1.2 (1.1,1.3)	1.3 (1.2,1.5)	0.0033
RII (95% CI)	0.3 (0.2,0.5)	0.5 (0.4,0.7)	0.7 (0.6,0.8)	0.7 (0.6,0.8)	0.0005
Coverage ratio(95% CI)					
Poorest (ref.)					
Poorer	1.2 (1.0,1.4)	1.2 (1.1,1.4)	1.0 (0.9,1.1)	1.0 (0.9,1.1)	0.0788
Middle	1.2 (1.0,1.4)	1.3 (1.1,1.5)	1.0 (0.9,1.1)	0.9 (0.8,1.0)	0.0030
Richer	1.4 (1.1,1.7)	1.3 (1.2,1.5)	1.1 (1.0,1.2)	1.1 (1.0,1.2)	0.0175
Richest	1.5 (1.3,1.8)	1.4 (1.2,1.6)	1.1 (1.0,1.2)	1.1 (0.9,1.2)	0.0019
RII (95% CI)	0.6 (0.5,0.8)	0.7 (0.5,0.8)	0.9 (0.8,1.0)	0.9 (0.8,1.0)	0.0026
Concentration Index (95% CI)	0.21 (0.12–0.28)	0.20 (0.12–0.29)	0.08 (0.02–0.13)	0.054 (−0.01–0.12)	na

*P value for test of heterogeneity in log prevalence ratio or RII across years

na = not applicable

## Discussion

### The trend of full vaccination coverage

Our results show that full vaccination coverage steadily increased from 65.6% in 2001 to 87.0% in 2011, but decreased to 77.8% in 2016.The trend was significant over time. Further analysis of NDHS 2016 showed the main reason for the significant decline in full vaccination to be the decline in the percentage of infants who received the third dose of DPT containing vaccine from 91.7% in 2011 to 85.9% in 2016 [20]. The drop out in the third dose of DPT increased from 5% (95% CI 3.1–7.6) in 2011 to 11% (95% CI 8.8–13.8) in 2016. However, the coverage of the measles-containing vaccine, which is provided after the third dose of DPT, has increased from 88.0% in 2011 to 90.4% in 2016, suggesting a missed opportunity for vaccination of DPT3 during child’s future contact with the health system, possibly at nine months.

KC et al. reported similar findings among under-five infants where the decline in DPT3 and polio vaccine were the main contributor to the decrease in full vaccination coverage [10]. Phase-out of community health workers’ positions such as Village Health Workers might have affected immunization service utilization since direct communication through the household visit by health workers had a positive impact on immunization service utilization [21, 26]. However, the decline in full vaccination coverage, mainly driven by the decline in DPT3 coverage is difficult to interpret. The decline may be due to health system factors such as changes in the national immunization program and the introduction of new vaccines in different regions of the country (DPT-HepB-HiB, Pneumococcal conjugate vaccines (PCV), Inactivated Polio Vaccine (IPV), Measles-Rubella (MR), and Japanese Encephalitis (JE). Logistics and supply chain management issues after the introduction of new vaccines contributed to the decline in DPT3 coverage in South Africa in 2009 [27]. Other possible explanation could be due to mothers’ and their newborns’ temporary move to her mother’s house [28] around 10–14 weeks of childbirth and less familiarity with vaccination schedule, and place in the new place. Another reason may be people’s perception of not feeling the importance of three doses of vaccine after one or two doses of the same vaccine has already been received. It may also be associated with a reduced feeling of threat against polio [10] since DPT and polio are administered simultaneously at 6,10 and 14 weeks. However, this needs further investigation.

When analyzed among the pooled sample from all four NDHS years, residence in Province 2, no maternal education, and lower household wealth quintile were found to be associated with significantly lower coverage of full vaccination among infants. Despite geographical accessibility, Province 2 has the lowest women literacy in Nepal [29], health facilities had lower performance in child health care [30] and have socio-cultural barriers compared to other provinces of Nepal. Our findings of higher full vaccination coverage among infants from richer families and those born to mothers with higher educational status are consistent with studies from Ethiopia and Bangladesh [31, 32]. Women from wealthy families may be more likely to accept “modern/medical” services than their poor counterparts [31]. Similarly, mothers with higher educational status might be aware of the preventive role of immunization service compared to women with no education. A meta-analysis on the role of maternal education on childhood vaccination also showed that maternal education plays a more important role in lower-income countries than in high-income countries [33].

### Equity gap in full vaccination coverage

Increased full vaccination coverage from 2001 until 2011 and a recent decline in full vaccination coverage in 2016, overall showed a narrowing equity gap by wealth quintiles and maternal educational status. The absolute inequalities in full vaccination coverage saw a drop over time by educational status and wealth quintiles. The Relative Index of inequality also showed an improvement over time, both for wealth quintile and for maternal education between 2001 and 2016. A similar study among under-five children by KC et al. [10] used data from the DHSs carried out in 2001, 2006, 2011 and MICS 2014 showing that the poorest wealth quintile with the most significant improvement in vaccination coverage, from 58% in 2001 to 77.9% in 2014 while the wealthiest quintile showing a little improvement from 84.8 to 86.0%. The study also found improving the slope index of inequality for infants who received all vaccines improved from 0.070 (95% CI: 0.061–0.078) to 0.026 (95% CI: 0.013–0.039) and relative index of inequality from 1.13 to 1.0. The decreased equity gap (by wealth, education) can be attributed to the concentrated efforts of the GoN in collaboration with non-governmental organizations and the local community, to focus on hard-to-reach and disadvantaged populations [34].

A significant decline in full vaccination coverage in Nepal after 2011, albeit retention of equity gain among the infants from households with poorest wealth quintile and those with no education, puts Nepal’s immunization program far behind GoN’s target to fully immunize more than 90% of children by 2020 [3]. Nepal’s experience of the significant simultaneous decline in overall full vaccination coverage, together with a decline in pro-rich inequity, is similar to that experienced by the Central African Republic [12]. Case studies from 10 of 75 countdown countries selected for measuring progress against MDG 2015 targets also showed an increased coverage for interventions administered at lower levels of the health system, including immunization, along with reduced equity gaps and improvements in associated health outcomes during the MDG era [35].

### Policy, practice and research implications

Although this study did not investigate health system factors affecting service utilization, the findings from Acharya et al. ‘s analysis of much lower coverage of DPT3 compared to DPT1 and DPT2 [20] partly indicate health system’s weakness to retain service users. Furthermore, lower DPT3 coverage than measles coverage clearly indicates a missed opportunity to utilize children’s contact with the health system. First of all, text messages or reminders/recalls or other systems need to be in place to remind/follow up [36] parents to ensure full vaccination on time. Second, there should be a mechanism to provide missed vaccines when children come in contact with the health system for other services in the future.

A higher decline in full immunization observed among infants born to mothers from middle and the highest income quintiles may have different reasons. An earlier study from Nepal has shown that improving the quality of the vaccination program maybe even more important than improving access to it [26]. While improving access is essential to reach some sections of the population, improving service quality may be more important to retain wealthy families. Higher waiting time associated with staff shortage and increased number of new vaccines might have discouraged wealthy families to complete the full immunization. Therefore, a higher declining trend of full immunization coverage among middle and rich income quintiles needs an urgent investigation and timely action. As the coverage of full immunization starts to decline, the momentum of pro-poor equity gain may be reversed, and poor and disadvantaged groups will be most likely to be missed out. Altogether, these findings highlight the need to focus on reaching the poor without forgetting the richer sections.

Our analysis showed that although in decreasing trend, inequalities in full immunization coverage persist by maternal educational status and household wealth quintiles with service utilization concentrated among those in higher wealth quintiles and those with higher education. Furthermore, the recent decline in full immunization coverage after 2011 was higher among infants born to mothers with no education with respect to infants born to mothers with higher education, which led to a spike in crude difference from 14.3% in 2011 to 23.4% in 2016 (Table 4). Additionally, values of SII and RII were higher for maternal educational status compared to household wealth quintile for all DHS surveys indicating that there is a larger inequality by maternal education status than by household wealth status. Furthermore, absolute inequality declined significantly over time for wealth quintiles, compared to maternal educational status. Since maternal education was found to be a strong predictor of immunization uptake in Nepal [10] and other settings [37], these findings suggest that infants born to women with no or less education need special targeting to increase full immunization coverage. More importantly, all levels of governments in Nepal need to devise policies to promote girls’ educational status since it has multiple returns on social, health, and economic sectors [38].

### Strengths and limitationss

We used nationally representative data from the four most recent DHS surveys. Furthermore, we merged these datasets, which increased the power of our regression analyses. We also used both the absolute and relative measures of inequality for the analysis of equity gaps in full vaccination coverage. And, we did this for wealth quintile as well as for maternal education, which are strong determinants of full vacination coverage. However, the study has some limitations. Only six antigens administered during the infancy were considered for comparison of coverage over the years because some of the antigens recently introduced were not available during the period covered by earlier surveys conducted in 2001, 2006 and 2011. Additionally, no supply-side factors were studied since DHS data lacks health service-related data. Variables related to socio-cultural practices, social norms, and beliefs regarding vaccination were not available. Furthermore, we included income quintiles as a proxy measure of socioeconomic status. However, multiple aspects of poverty might reflect socioeconomic status better than income quintiles. When vaccination cards were not available, interviewers relied on mothers’ reports to determine receipt of vaccination . Therefore, misclassification could have arisen if mothers did not correctly recall the name and receipt of the vaccine.

## Conclusion

This study, utilizing data from four recent demographic and health surveys from Nepal, demonstrates an increase in full vaccination coverage from 2001 until 2011 and a recent decrease, moving away from the national target. Both the absolute and relative inequalities by maternal education and wealth quintile decreased from 2001 to 2016. Altogether, these findings imply a need to redirect the focus of the national immunization program towards an overall increase in vaccination coverage in the country, with a greater focus among mothers with less education, poorer wealth quintile, and those living in Province 2.

## Data Availability

Data can be easily available through the DHS program website (www.dhsprogram.com) upon the request.

## Abbreviations

AOR: Adjusted odds ratio
BCG: Bacille calmette guerin
CI: Confidence interval
DHS: Demographic and health survey
DPT: Diphtheria, pertussis and tetanus
GoN: Government of nepal
IPV: Inactivated polio vaccine
JE: Japanese encephalitis
MICS: Multiple indicators cluster survey
MR: Measles-rubella
NDHS: Nepal demography and health survey
NHRC: Nepal realth research council
NIP: National immunization program
PCV: Pneumococcal conjugate vaccine
Q1: First quintile
Q5: Fifth quintile
RII: Relative index of inequality
SES: Socioeconomic status
SII: Slope index of inequality
